# Mesenchymal stem cells express epidermal markers in an *in vitro* reconstructed human skin model

**DOI:** 10.3389/fcell.2022.1012637

**Published:** 2023-01-12

**Authors:** Jeniffer Farias Dos Santos, Bruna Letícia Freitas-Marchi, Gustavo Roncoli Reigado, Silvia Romano de Assis, Silvya Stuchi Maria Engler, Felipe Santiago Chambergo Alcalde, Viviane Abreu Nunes

**Affiliations:** ^1^ Laboratory of Skin Physiology and Tissue Bioengineering, School of Arts, Sciences and Humanities, University of Sao Paulo (EACH-USP), São Paulo, São Paulo, Brazil; ^2^ Skin Biology Group, iNOVA Pele, School of Pharmaceutical Sciences (FCF), University of São Paulo, São Paulo, São Paulo, Brazil

**Keywords:** mesenchymal stem cells, skin, epidermal differentiation, keratinocytes, organotypic cultures

## Abstract

**Introduction:** In skin traumas, such as burns, epidermal homeostasis is affected, often requiring clinical approaches. Different therapeutic strategies can be used including transplantation, besides the use of synthetic or natural materials with allogeneic cells. In this context, tissue engineering is an essential tool for skin regeneration, and using mesenchymal stem cells (MSC) from the umbilical cord appears to be a promising strategy in regenerative medicine due to its renewal and differentiation potential and hypo immunogenicity. We evaluated the transdifferentiation of MSC from umbilical cord into keratinocytes in three-dimensional (3D) *in vitro* skin models, using dermal equivalents composed by type I collagen with dermal fibroblasts and a commercial porcine skin decellularized matrix, both cultured at air-liquid interface (ALI).

**Methods:** The expression of epidermal proteins cytokeratins (CK) 5, 14 and 10, involucrin and filaggrin was investigated by real-time PCR and immunofluorescence, in addition to the activity of epidermal kallikreins (KLK) on the hydrolysis of fluorogenic substrates.

**Results and discussion:** The cultivation of MSCs with differentiation medium on these dermal supports resulted in organotypic cultures characterized by the expression of the epidermal markers CK5, CK14, CK10 and involucrin, mainly on the 7^th^ day of culture, and filaggrin at 10^th^ day in ALI. Also, there was a 3-fold increase in the KLK activity in the epidermal equivalents composed by MSC induced to differentiate into keratinocytes compared to the control (MSC cultivated in the proliferation medium). Specifically, the use of collagen and fibroblasts resulted in a more organized MSC-based organotypic culture in comparison to the decellularized matrix. Despite the non-typical epithelium structure formed by MSC onto dermal equivalents, the expression of important epidermal markers in addition to the paracrine effects of these cells in skin may indicate its potential use to produce skin-based substitutes.

## Introduction

Tissue engineering is an important strategy for treating skin traumas and generating skin substitutes and adjuvants that can accelerate epidermal reconstitution (Nourian Dehkordi et al., 2019). Primary keratinocytes have been used in the skin *in vitro* 3D models aiming for their use in tissue engineering (Iljas et al., 2021; Smith et al., 2022). However, their limited proliferative and expansion capacity suggests using other cellular sources such as stem cells.

An important source of stem cells is the human umbilical cord (Wang et al., 2004; Santos et al., 2019) formed by hematopoietic and mesenchymal components (MSC, mesenchymal stem cells). The harvest of these cells involves a simple, safe and painless procedure, depending only on the maternal consent, which is an advantage over other sources of adult stem cells (Santos et al., 2019). Specifically, these cells are immunologically more immature than other sources, resulting in a lower frequency of graft-versus-host disease (Martin-Piedra et al., 2019).

Studies about the potential of umbilical cord mesenchymal stem cells (MSC) have shown that these cells are able to differentiate into several cell types (Nunes et al., 2007; Santos et al., 2019; Stefańska et al., 2020). We have already demonstrated that MSCs from umbilical cord are able to differentiate into keratinocytes under 2D culturing with epidermal growth factor (EGF) (Santos et al., 2019). During this process, it was verified the expression of the cytokeratins (CK) and involucrin, as well as the enzymatic activity of tissue kallikreins (KLK), proteolytic enzymes present in the most differentiated layers of the epidermis.

Despite many advances in the use of MSC for the establishment of skin 3D models or for the treatment of dermatological diseases, the results are still insufficient to mimic the skin microenvironment in a functional model, in which epidermal markers are expressed in a temporal relationship to that which occurs *in vivo* (Kamolz et al., 2006; Schneider et al., 2010; Toai et al., 2011; Tam et al., 2014; Chen et al., 2015; Steffens et al., 2015; Xie et al., 2016). In this sense, the differentiation of keratinocytes has been studied in different extracellular matrix (ECM)-based supports that are able to create a microenvironment favorable for skin regeneration, tissue remodeling and functional repair (Yi et al., 2017).

We have addressed here the use of MSC on dermal supports in order to evaluate the feasibility to incorporate them in skin models for tissue engineering or wound healing. The culture of MSC on these dermal supports resulted in a stratified organotypic culture characterized by the expression of the specific epidermal markers CK5, 14, and 10, involucrin, filaggrin and epidermal kallikreins. The knowledge about MSC behavior in human *in vitro* skin models may provide useful tools for the study of skin biology that are essential for the development of therapeutic strategies for skin pathologies and traumas (Bian et al., 2022; Capilla-González et al., 2022).

## Materials and methods

### Cell cultures

MSC from the umbilical cord, stored in the Cell Bank of the Laboratory of Skin Physiology and Tissue Bioengineering of School of Arts, Sciences and Humanities of University of São Paulo (EACH-USP), were used in accordance with the Ethics Committee of the University of São Paulo (Number 4.351.554). After thawing, cells were plated in 75 cm^2^ flasks at a density of 5 × 105 cells/mL and incubated in a humidified atmosphere, 5% CO_2_, at 37°C in Dulbecco’s modified Eagle’s medium (DMEM) with low glucose concentration (2 mM) and 10% fetal bovine serum (FBS), named LD10, and called proliferation medium.

Human primary dermal fibroblasts from a 10 months-old male in first passage (code nh-skp-FB0050) and human basal keratinocytes from a 4 years-old female in second passage (code nh-skp-KT0085) were obtained from Rio de Janeiro cell bank (BCRJ). These cells were isolated from the epidermal and dermal layer, respectively, of human neonatal foreskin. Fibroblasts were cultured with LD10 using the same conditions as MSC. The keratinocytes were maintained in Keratinocyte Growth Medium (KGM) with its respective supplement (KBM™ Gold™ Basal Medium and KGM™ Gold™ SingleQuots™, according to manufacturer instructions, Lonza^®^) under a humidified atmosphere, with 5% of CO_2_ at 37°C.

### Skin organotypic cultures using dermal equivalents

To skin *in vitro* reconstruction, the air-liquid interface (ALI) technique was used, in which the dermal equivalent is in contact with the culture medium, and the epidermal equivalent is in contact with the air. The culture medium was changed every 48 h and the samples were analyzed in different periods of culture in ALI. Two different approaches were used to mimic the dermis: rat tail type I collagen (Corning Costar) with fibroblasts and the porcine decellularized skin matrix MatriXpec™ (TissueLabs™). For the dermal equivalent of collagen, human dermal fibroblasts (1.5 × 105/dermal equivalent) were resuspended in 3.0 mg/mL type I collagen solution. This solution was transferred to a 24-well plate and kept in an incubator at 37°C for 20 min. The plate was kept for 2 h at 37°C in a humidified atmosphere with 5% CO_2_. For the decellularized skin matrix MatriXpec, dermal fibroblasts (1.5 × 105/dermis) were resuspended in 1 mL of the commercial product and incubated at 37°C in a humidified atmosphere with 5% CO_2_ for gelatinization.

Epidermal equivalents were composed of 2.5 × 105 cells/epidermis: basal keratinocytes (positive control) were cultured in KGM and MSC were cultured in LD10 (proliferation medium) or KGM. For epidermis assembly, cells were first immersed, for 1 day, in KGM/DM (1:1) (DM, differentiation medium) and then exposed to ALI for 10 days in KGM/DM medium only facing the bottom of the dermal equivalent. DM was prepared by adding DMEM and Ham’s-F12 medium (GibcoBRL, Gaithersburg, MD) (3:1), 10% FBS and the supplements 10.1 nM cholerae toxin, 5 μg/mL insulin, 5 μg/mL apo-transferrin, 0.4 μg/mL hydrocortisone 21-hemisuccinate, 1 ng/mL epidermal Growth Factor (EGF), according to Catarino et al. (2018). MSC cultured with proliferation medium were used as negative control for *in vitro* skin reconstruction. The culture medium was changed every 48 h.

### Histological analysis of organotypic cultures

After the period of exposure to ALI of 10 days, the samples were fixed in 10% formalin buffered in PBS at 4°C for 4 h, dehydrated in increasing concentrations of alcohols and embedded in paraffin. The blocks were then cut on a Leica RM2165 automatic microtome (Leica, Wetzear, Germany) producing cross-sections of approximately 5 µm. The sections were placed on histological slides and dried in an oven at 60°C for 2 h to remove paraffin excess. Then, the samples were diaphanized in 100% xylene for 10 min, hydrated in increasing alcohol content (100%, 95%, 85%, and 70% ethanol), washed in distilled water for 5 min, and stained with hematoxylin-eosin (HE). Cover slides were mounted with Permount^®^ (Fisher Scientific, Pittsburgh, United States). The microscopic images were obtained using an Olympus BX 60 microscope with an attached digital camera (Axio CAM HRc—Zeiss^®^).

### RNA extraction and synthesis of complementary DNA strand

After 4, 7, and 10 days of ALI, the organotypic cultures were collected and washed with PBS. For RNA extraction, the Mini Kit RNeasy (Qiagen, Frederick, MD, United States) was used, according to the manufacturer instructions. The samples were placed on the column containing a silica membrane, lysed with RLT buffer and homogenized. Ethanol was then added to the lysate, creating the conditions that promote selective binding of RNA to the membrane. Contaminants were washed in RW1 buffer, and RPE buffer and then the RNA was eluted in Ultra-pure water. After quantification, the complementary strand of DNA (cDNA) was synthesized from 2 ng of RNA using the RT2 First Strand kit (Qiagen, Frederick, MD, United States) in two steps: the first consisted of eliminating the genomic DNA using 8 μL of RNA (2 μg) and 2 μL of ×5 concentrated GE buffer, the mixture being incubated for 5 min at 42°C; the second step refers to reverse transcription, in which 10 µL of the product from the first step, 4 µL of 5x concentrated reaction buffer (BC3), 1 µL of P2 control (oligo dT) and dNTPs were used 10 mM, in a final volume of 20 µL. This solution was incubated for 15 min at 42°C and then for 5 min at 95°C in a thermocycler (Eppendorf, United States), according to the manufacturer recommendations.

### Quantitative PCR in real-time (qPCR)

To quantify the expression of CK14, CK10, involucrin, filaggrin, e-cadherin and vimentin, the quantitative PCR technique was used using the SYBR^®^ green PCR Master Mix (ThermoFisher Scientific, Massachusetts, United States) and the following program: 95°C for 10 min, 40 cycles of 95°C for 30 s, 60°C for 45 s and 72°C for 1 min. The final step was performed at 72°C for 10 min, according to the manufacturer instructions. The expression of the genes of interest was normalized by the expression of the glyceraldehyde 3-phosphate dehydrogenase (GAPDH) gene. Dermal fibroblasts, MSC and keratinocytes two-dimensionally cultured, in addition to the control skin formed by stratified keratinocytes, were also analyzed for the expression of these proteins. The expression was analyzed in relation to the epidermis formed with MSCs grown in LD10 (proliferation medium), used as a negative control. The oligonucleotides were designed, according to Table 1, in order to avoid non-specific annealing and respecting the recommendations for use in quantitative PCR. Reactions were performed on the Eco™ Real-Time PCR System (Illumina, San Diego, CA, United States).

**TABLE 1:** Sequences of oligonucleotides used in qPCR experiments.

Prime	Sequence	Reference
CK10	Forward: CAACCTAACAACTGATAATGCC	Santos et al. (2019)
	Reverse: GTCTTTCATTTCCTCCTCGT	
CK14	Forward: GGAACAAGATTCTCACAGCC	Santos et al. (2019)
	Reverse: TCCATCTCCACATTGACATCTC	
Involucrin	Forward: AATGAAACAGCCAACTCCACTGCC	Fernandes et al. (2022)
	Reverse: TCTTGCTTTGATGGGACCTCCACT	
Filaggrin	Forward: TGAAGCCTATGACACCACTGA	Wang et al. (2017)
	Reverse: TCCCCTACGCTTTCTTGTCCT	
GAPDH	Forward: CATTTCCTGGTATGACAACGA	Santos et al. (2019)
	Reverse: GCACAGGGTACTTTATTGATGG	
E-cadherin	Forward: CCCACCACGTACAAGGGTC	Stewart et al. (2012)
	Reverse: CTGGGGTATTGGGGGCATC	
Vimentin	Forward: GAGTCCACTGAGTACCGGAGAC	Sudo et al. (2013)

### Expression of the epidermal markers CK5, CK10 and involucrin by immunofluorescence

After deparaffinization and hydration, the slides were washed with PBS, permeabilized with PBS containing 0.1% Triton X-100 for 20 min and washed with PBS. Antigen retrieval was performed by incubating the slides in 0.01 M citrate buffer pH 6.0 for 30 min at 95°C. Non-specific sites were blocked by the incubation of samples with PBS containing 10% FBS for 20 min. Then, the primary antibodies anti-CK10, anti-CK5 and anti-involucrin were added (1:100), protected from light for 12 h at 4°C. For visualization of positively labeled cells, FITC-conjugated anti-IgG secondary antibodies were used. Slides were washed and analyzed under a fluorescence microscope (Zeiss, Germany). The pictures are representative of images analyzed in blinded tests, with *n* ≥ 3 in at least 10 random fields registered under the same exposure. The images were analyzed using image J software (https://imagej.nih.gov/ij/; Schneider et al., 2012). The mean fluorescence intensity per pixel was determined, and the threshold of 10 was defined as background.

### Activity of epidermal kallikreins

To evaluate the enzyme activity of epidermal kallikreins, the dermal substitutes, consisting of collagen with fibroblasts or MatriXpec, were initially dissociated from the epidermis by mechanical separation, and the samples were lysed with 200 μL of 25 mM HEPES buffer, pH 7.5, 0.5% Triton X-100 (lysis buffer) producing extracts. The epidermis formed by basal keratinocytes was analyzed after 4, 7, and 10 days of ALI, while the epidermis potentially formed by MSC was analyzed only after 10 days of ALI. The activity of KLK in extracts was verified on the hydrolysis of fluorogenic substrates (FRET) (Angelo et al., 2006; Loura, 2011) suitable to detect KLK5 (Abz-KLRSSKQ-EDDnp), KLK6 (Abz-AFRFSQ-EDDnp) and KLK7 (Abz-KLYSSKQ-EDDnp) activities. 40 μL of samples were used in 50 μL of 2x concentrated enzyme reaction buffer (100 mM Tris, pH 8.0, 300 mM NaCl, and 0.01% Tween 20) and 10 μL of substrate (100 μM), in a final volume of 100 μL. Substrate hydrolysis (10 μM) was accompanied by an increase in fluorescence at λex = 320 nm and λem = 420 nm in the Synergy HT plate reader (Biotek, Winooski, VT, United States) according to Santos et al. (2019). Mean rate results for each reaction were obtained using Gen5™ software (BioTek, Winooski, VT, United States). Enzyme activity was measured in arbitrary fluorescence units (AFU) per min and expressed in terms of the mean reaction rate per 1 mg of protein present in the extracts.

### Statistical analysis

Quantitative data were expressed as mean values ± standard error (SE) with *n* ≥ 3. For statistical comparison, the results were analyzed by the GraphPad Prism 5 software, using the two-way ANOVA plus Bonferroni post-test, with statistically significance of *p* < .05 (*); *p* < .01 (**), *p* < .001 (***) from the control. Under microscopy, the morphology of the cells, their three-dimensional arrangement and possible epidermal stratification were evaluated using the image J software (Schneider et al., 2012).

## Results

### Histological analysis of organotypic cultures

**FIGURE 1:**
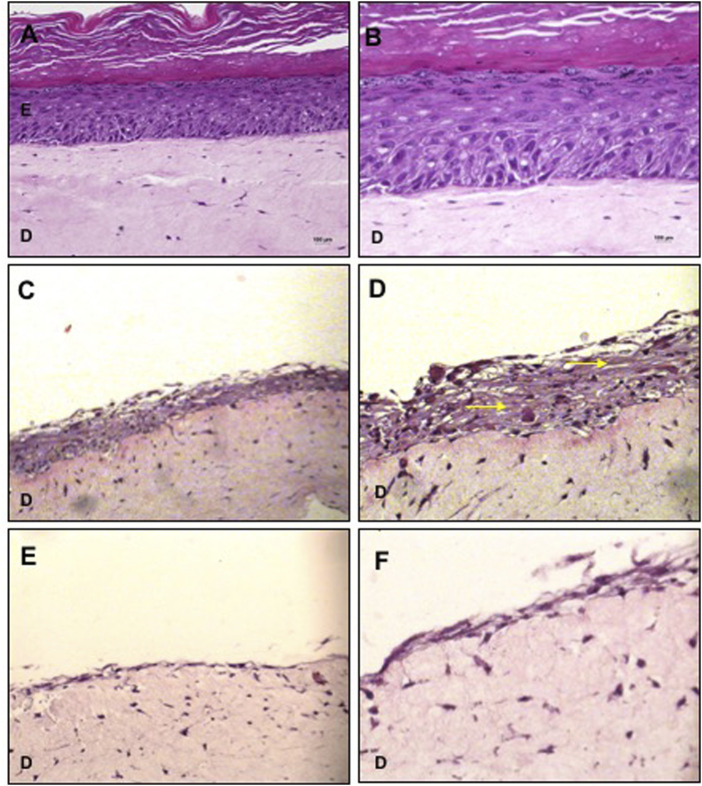
Organotypic cultures using collagen with fibroblasts as dermal equivalent. **(A,B)** Basal keratinocytes cultured with KGM/DM were used as positive control for the stratified epidermis. **(C,D)** MSC cultured with KGM/DM or with **(E,F)** LD10 medium. After 1 day of immersion in the indicated medium in each case, cultures were exposed to ALI for 10 days in the DM **(A,D)** or LD10 medium **(E,F)**. Hematoxylin-eosin staining. Magnification of 200x **(A, C, and E)** and 400x **(B, D, and F)**. Yellow arrows: extracellular matrix/intercellular substance. Scale bar: 100 µm.

The cultivation of basal keratinocytes onto the dermal equivalent constituted by collagen with fibroblast resulted in a typical stratified epidermis I with all well-defined layers (Figures 1A, B), confirming that 10 days of culture in ALI, under the specified conditions, were sufficient for terminal differentiation of keratinocytes, being considered as a positive control.

MSCs cultured for 1 day with KGM/DM and exposed to ALI for 10 days proliferated and formed a stratified organotypic culture composed by cells distributed in a basophilic intercellular substance (Figures 1C, D). In these samples, the dermo epidermal separation was not clear and the presence of unstained vacuoles was verified. MSC grown with LD10 were arranged as a monolayer over the dermal equivalent (Figures 1E, F) and their cultivation resulted in the lowest epidermal thickness compared to the differentiation medium.

**FIGURE 2:**
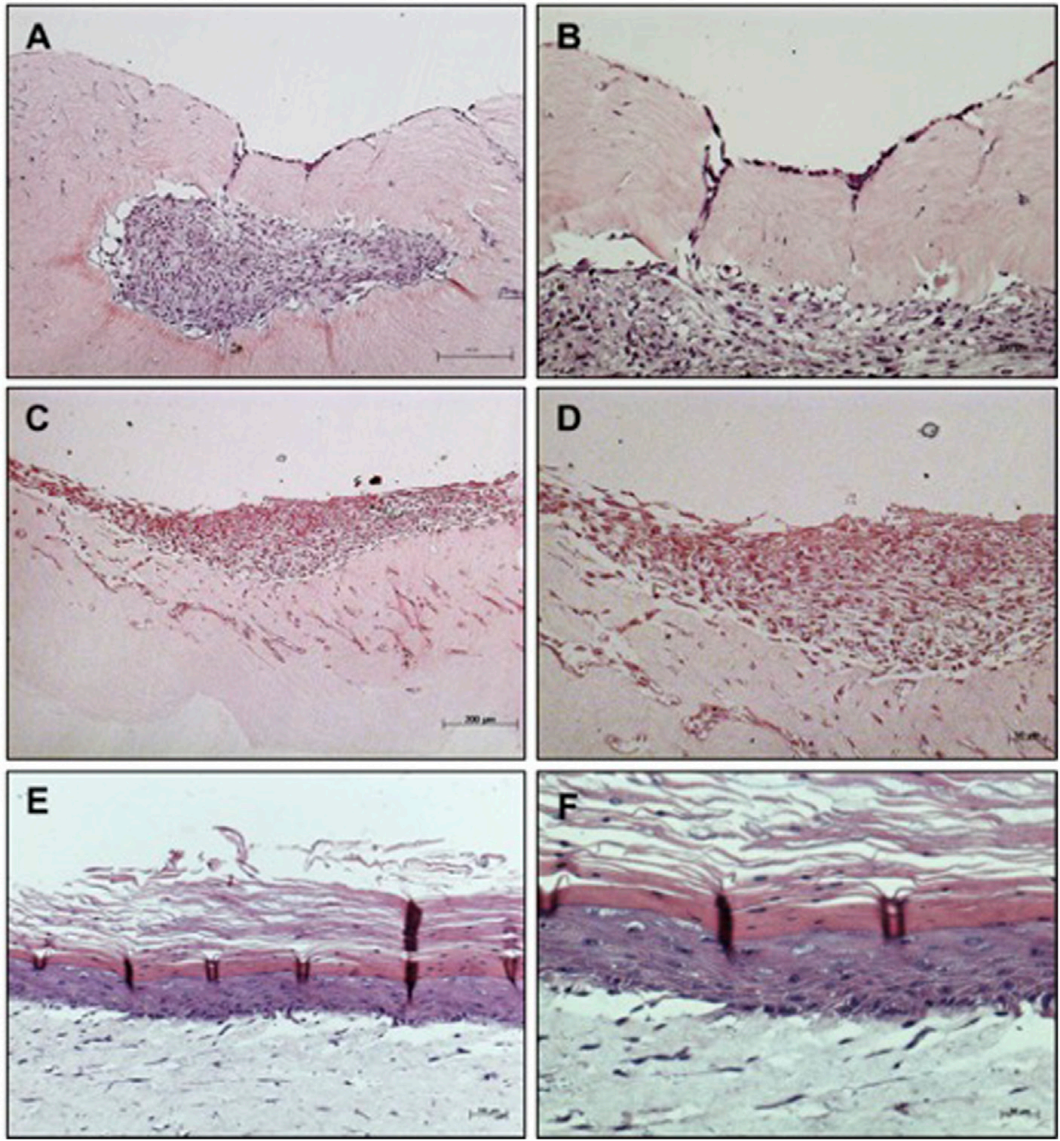
Organotypic cultures using MatriXpec™ as dermal equivalent. **(A,B)** MSC cultured with LD10 or **(C,D)** MSC cultured with KGM/DM **(E,F)** Basal keratinocytes cultured with KGM/DM were used as positive control for the stratified epidermis using MatriXpec. After 1 day of immersion in the indicated medium in each case, cultures were exposed to ALI for 10 days in **(A,B)** LD10 medium or KGM/DM **(C–F)**. Hematoxylin-eosin staining. Magnification of ×100 **(A, C, and E)** and 200x **(B, D, and F)**.

Macroscopically, MSC grown on MatriXpec™ with LD10, unlikely it was observed for the dermal equivalent of collagen with fibroblasts, promoted an intense contraction of the dermal support suggesting that MSC exhibited great interaction with this material. Also, the cells showed exacerbated migration/proliferation towards the dermal equivalent (Figures 2A, B), when compared to MSC cultured onto collagen (Figures 1C–F) resulting in some cases in the complete wrapping of MSCs by the matrix. This phenomenon was not observed when cells were cultured with KGM/DM (Figures 2C, D). In this substrate, MSCs are able to proliferate and form layers resulting in a stratified organotypic culture with no typical epithelial phenotype, similar to the observed when MSC were grown onto the dermal equivalent of collagen with fibroblasts. The cultivation of basal keratinocytes onto MatriXpec resulted in a typical stratified epidermis (Figures 2E, F) similar to the observed when these cells were cultured onto the collagen support.

### Expression of epidermal markers by qPCR

**FIGURE 3:**
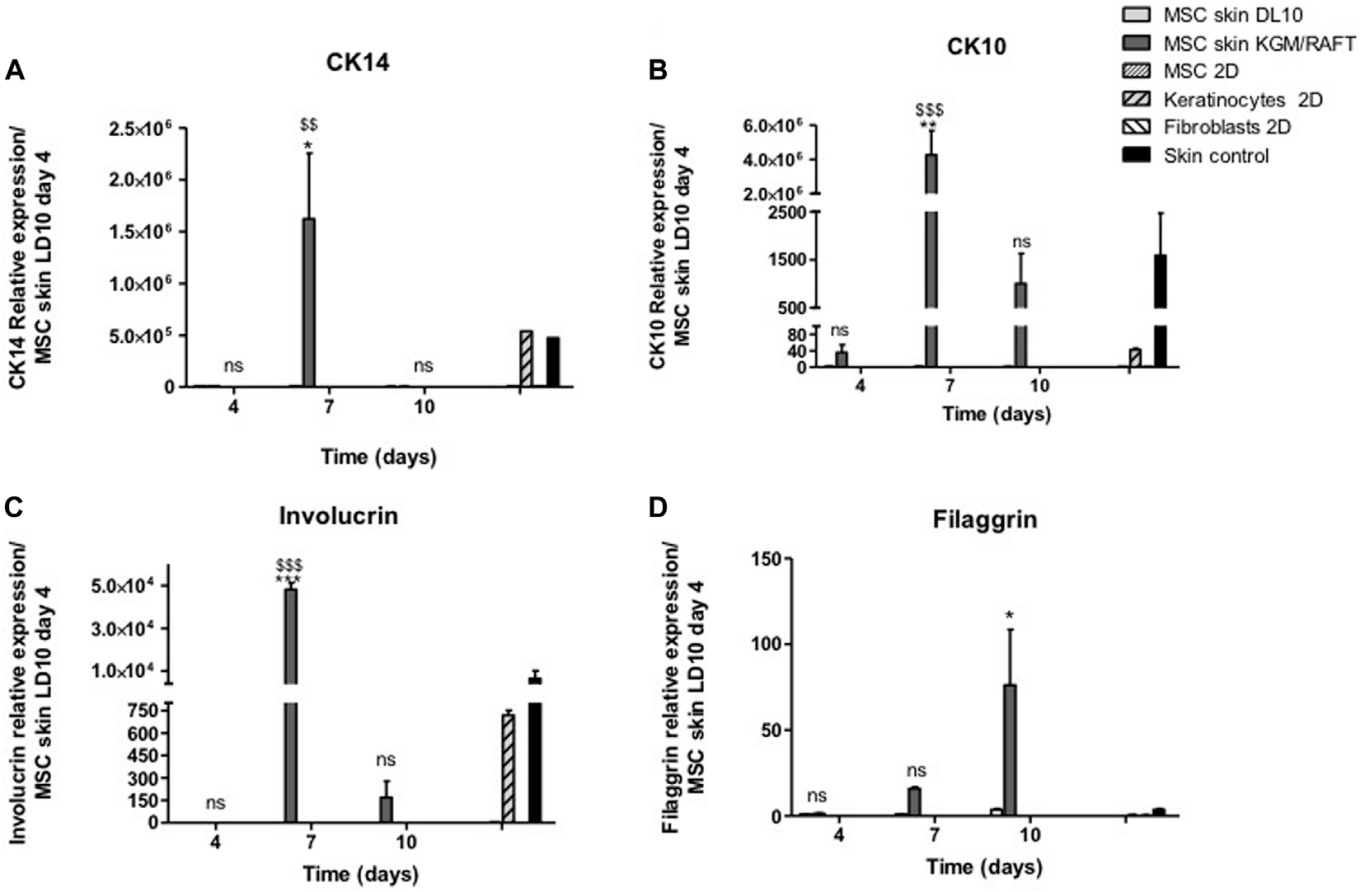
Expression of epidermal markers in the organotypic skin cultures. **(A)** CK14, **(B)** CK10, **(C)** involucrin, and **(D)** filaggrin. MSCs were cultivated with LD10 or KGM/DM, during 4, 7, and 10 days, onto the collagen matrix. MSCs and fibroblasts cultured in 2D were negative controls, and keratinocytes cultured in 2D and control skin were positive controls. Data was normalized by the expression of the constitutive gene GAPDH and analyzed in relation to MSCs used as epidermal equivalent and cultivated with LD10 on the fourth day of exposure to ALI. * (*p* < .05) or ** (*p* < .01), different in comparison to cultivation with LD10 during the same period. $$ (*p* < .01) or $$$ (*p* < .001), different in comparison to MSC cultivated with LD10 at day 4; ns: not significant.

The expression of CK14 and CK10, present in the basal and suprabasal strata of the epidermis, respectively, was evaluated after different periods of exposure to ALI. The expression of CK14 was verified on the 7th day of culture in the potential epidermis obtained with MSCs cultivated with KGM/DM, with no significant expression being observed in the other analyzed periods (Figure 3A), when compared to MSCs cultivated in LD10 at the same day of ALI. The presence of this protein was not observed in MSCs and fibroblasts cultured with LD10 in 2D systems whereas basal keratinocytes both in 2D and 3D systems (control skin) showed CK14 expression.

After 7 days of ALI, there was an increase in the expression of CK10 in skins formed by MSCs induced to differentiation. On the 10th day of culture in ALI, it is still possible to observe the significant expression of this protein, which is absent in MSCs cultivated with LD10 for the same period (Figure 3B). The expression verified on the 4th day of culture was similar to the expression in basal keratinocytes, suggesting the commitment of MSCs to the epidermal lineage at the beginning of the culture period, which did not occur in MSCs and fibroblasts cultured with LD10.

Involucrin expression was also investigated after 4, 7, and 10 days of exposure to ALI. Similar to CK10, involucrin had its expression increased on the 7th day in potential skins formed with MSC induced to differentiation compared to organotypic cultures formed MSC cultivated with LD10 (Figure 3C). 2D-cultured basal keratinocytes showed involucrin expression 16.3 times lower than the control skins while 2D-cultured MSC in LD10 did not show involucrin expression.

Filaggrin expression presented a different expression profile in comparison to the other analyzed proteins showing an increase at the end of the cultivation period (10 days) in MSCs induced to differentiation (Figure 3D). 2D-cultured basal keratinocytes showed lower expression (8.2-fold) of filaggrin than control skin, and MSC dimensionally cultured in LD10 did not show filaggrin expression, as expected.

**FIGURE 4:**
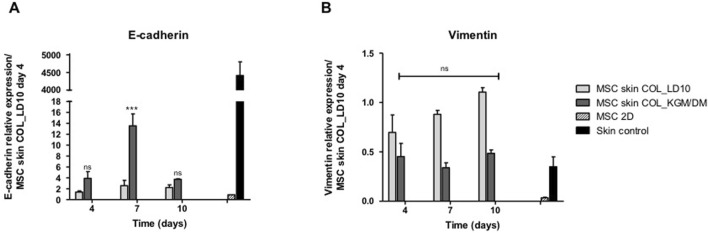
Expression of e-cadherin and vimentin. **(A)** E-cadherin and **(B)** Vimentin. MSCs were cultivated with LD10 or KGM/DM, during 4, 7, and 10 days onto collagen. MSC cultured in 2D was the negative control, and control skin was the positive control. Data was normalized by the expression of the constitutive gene GAPDH and analyzed in relation to MSCs used as epidermal equivalent and cultivated with LD10 on the fourth day of exposure to ALI. * (*p* < .05), *** (*p* < .001) different in comparison to MSC cultivated with LD10 at day 4; ns: not significant.

The expression of those epidermal markers was accompanied by E-cadherin up regulation (Figure 4A) and vimentin down regulation (Figure 4B), suggesting the transition to epithelial phenotype and loss of the mesenchymal marker vimentin by MSC induced to epidermal differentiation.

### Expression of the epidermal markers CK5, CK10 and involucrin by immunofluorescence

**FIGURE 5:**
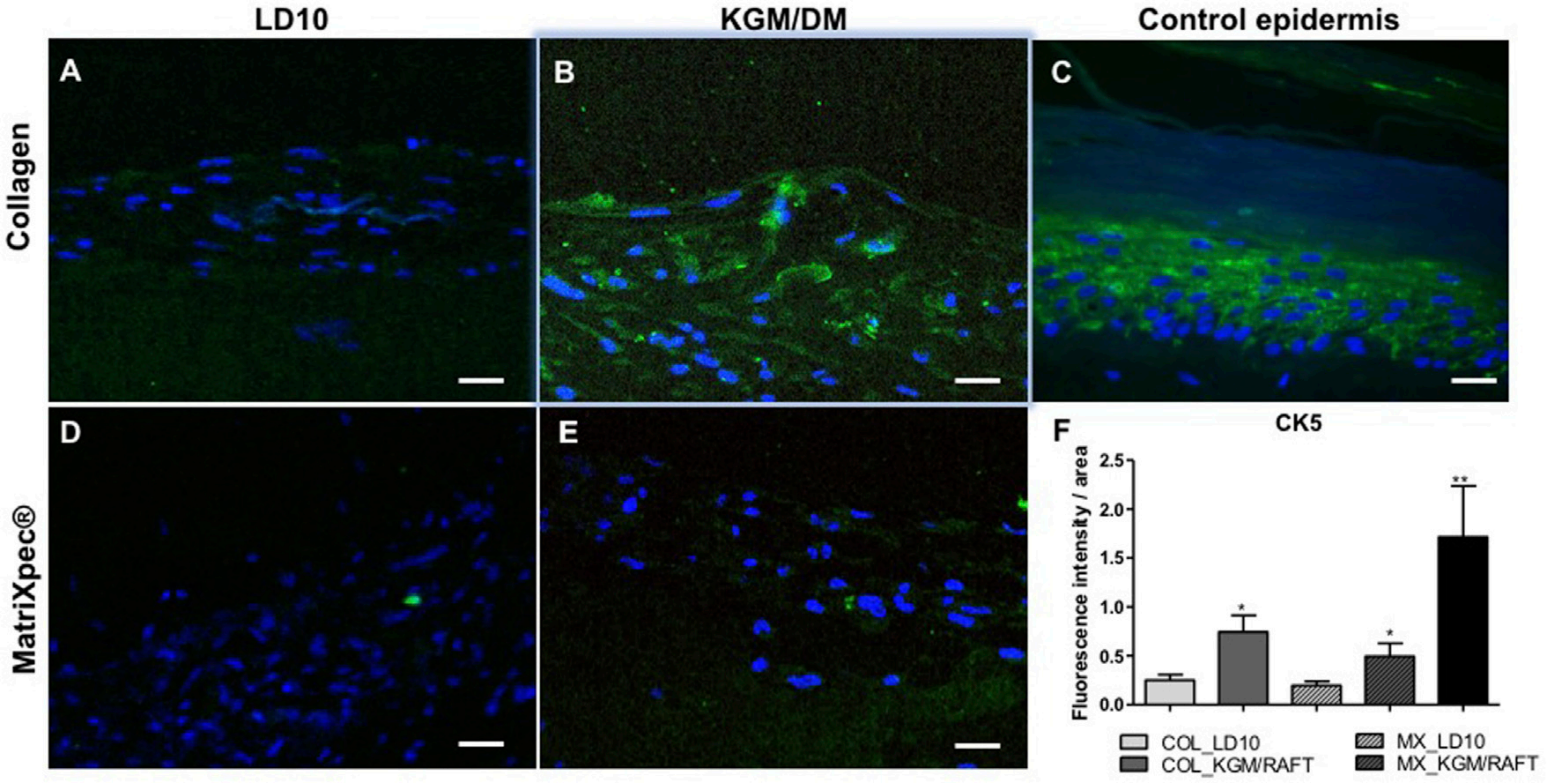
Expression of CK5 by immunofluorescence. **(A,D)** MSC cultured with LD10 or **(B,E)** KGM/DM onto **(A,B)** collagen or **(D,E)** MatriXpec™. **(C)** Skin formed by keratinocytes (control). CK5 labeling is shown in green. Nuclei are stained in blue (DAPI Magnification of 200x. Scale bar: 100 µm. **(F)** Mean fluorescence intensity per pixel in each group was compared to organotypic cultures formed by MSC cultivated onto collagen support with LD10 (COL_LD10). * (*p* < .05) and ** (*p* < .01).

After 10 days of exposure to ALI, the expression of different epidermal markers was analyzed by immunofluorescence allowing access to the localization of those proteins in the potential reconstructed skin. MSCs grown in LD10 onto collagen with fibroblasts or MatriXpec™ were not positively labeled for CK5 (Figures 5A–D), a protein found in the basal layer of the epidermis (Figure 5C). This protein was only identified in cells induced to differentiate onto collagen (Figure 5B), although it was not specifically related to an epidermis stratum. Specifically, the expression of CK5 was barely verified when the cells were cultured with KGM/DM onto the MatriXpec™ support (Figure 5E). The mean fluorescence intensity per group is shown in the Figure 5F, in agreement with the visual detection.

**FIGURE 6:**
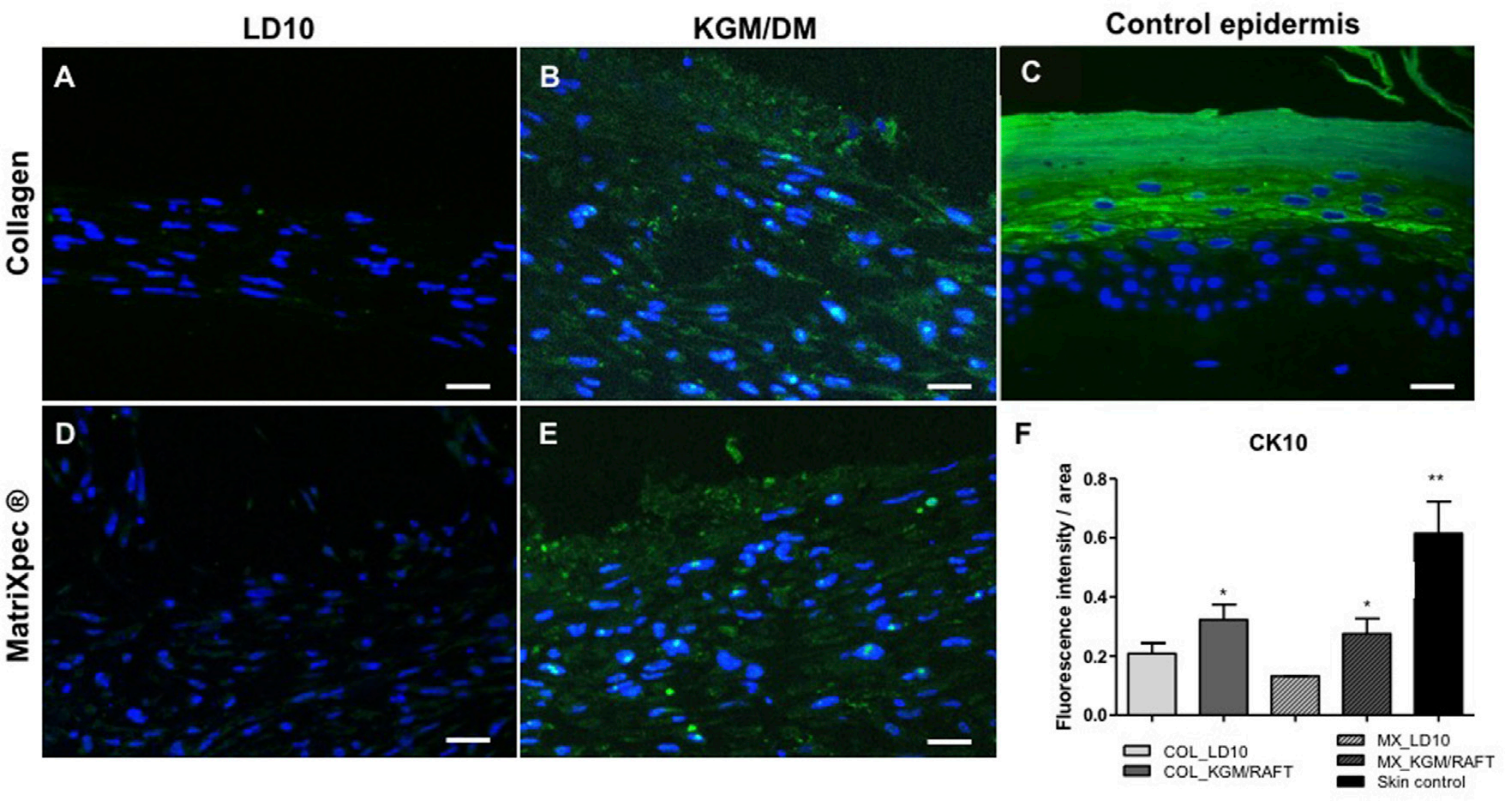
Expression of CK10 by immunofluorescence. **(A,D)** MSC cultured with LD10 or **(B,E)** KGM/DM onto **(A,B)** collagen or **(D,E)** MatriXpec™. **(C)** Skin formed by keratinocytes (control). CK10 labeling is shown in green. Nuclei are stained in blue (DAPI Magnification of 200x. Scale bar: 100 µm. **(F)** Mean fluorescence intensity per pixel in each group was compared to organotypic cultures formed by MSC cultivated onto collagen support with LD10 (COL_LD10). * (*p* < .05) and ** (*p* < .01).

Regarding CK10, a protein expressed in the suprabasal layers of the epidermis as identified in the control epidermis (Figure 6C), it is possible to observe the expression of this protein randomly distributed in the organotypic culture formed by MSCs induced to differentiation with KGM/DM using both matrices as dermal support (Figures 6D, E). Nevertheless, CK10 was barely detected when MSCs were cultured with LD10 onto collagen or MatriXpec™ supports (Figures 6A–D, respectively), as demonstrated by the fluorescence quantification (Figure 6F).

**FIGURE 7:**
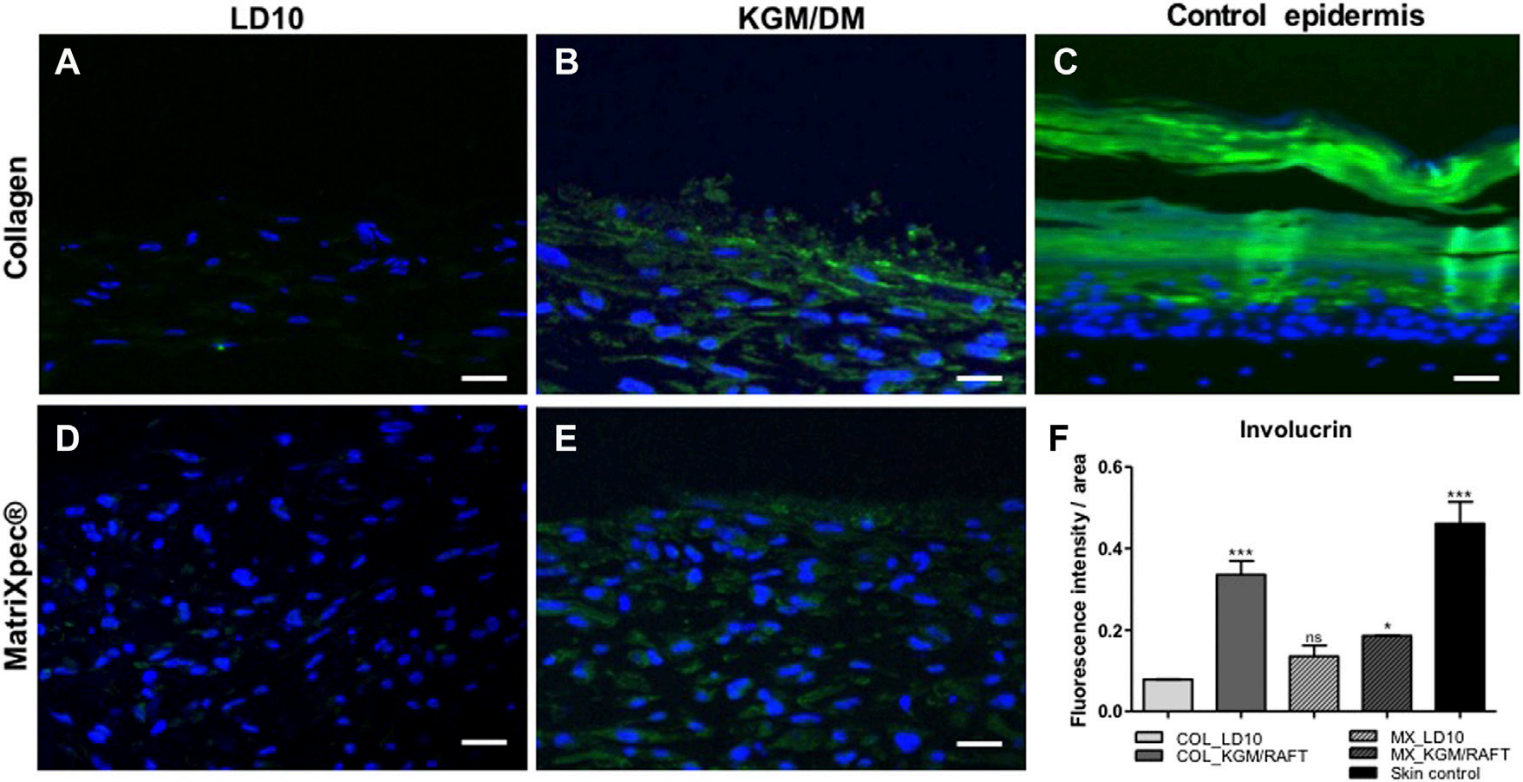
Expression of involucrin by immunofluorescence. **(A,D)** MSC cultured with LD10 or **(B,E)** KGM/DM onto **(A,B)** collagen or **(D,E)** MatriXpec™. **(C)** Skin formed by keratinocytes (control). Involucrin labeling is shown in green. Nuclei are stained in blue (DAPI Magnification of ×200. Scale bar: 100 µm. **(F)** Mean fluorescence intensity per pixel in each group was compared to organotypic cultures formed by MSC cultivated onto collagen support with LD10 (COL_LD10). * (*p* < .05) and *** (*p* < .001).

Similarly observed for CK10, involucrin, a protein expressed in the terminal differentiated layers of the epidermis (Figure 7C), was not identified when MSC were cultured with LD10, either onto collagen (Figure 7B) or (Figure 7D) MatriXpec™ dermal equivalents. However, under differentiation onto the collagen matrix (Figure 7C), cells showed involucrin expression, which was verified mainly in the upper layers of the organotypic culture. The expression was also detected in cells differentiated on MatriXpec™, but involucrin positive cells were distributed along the potential epidermis without a characteristic arrangement (Figure 7E). The fluorescence intensity per group is shown in the Figure 7F, being possible to observe involucrin expression, at least, 3-fold higher in the organotypic cultures of MSC induced to differentiation onto collagen support in comparison to the ones cultivated with LD10 (control). This difference was less marked between MSC cultivated onto MatriXpec™ with KGM/DM or LD10.

### Detection of activity of epidermal kallikreins

**FIGURE 8:**
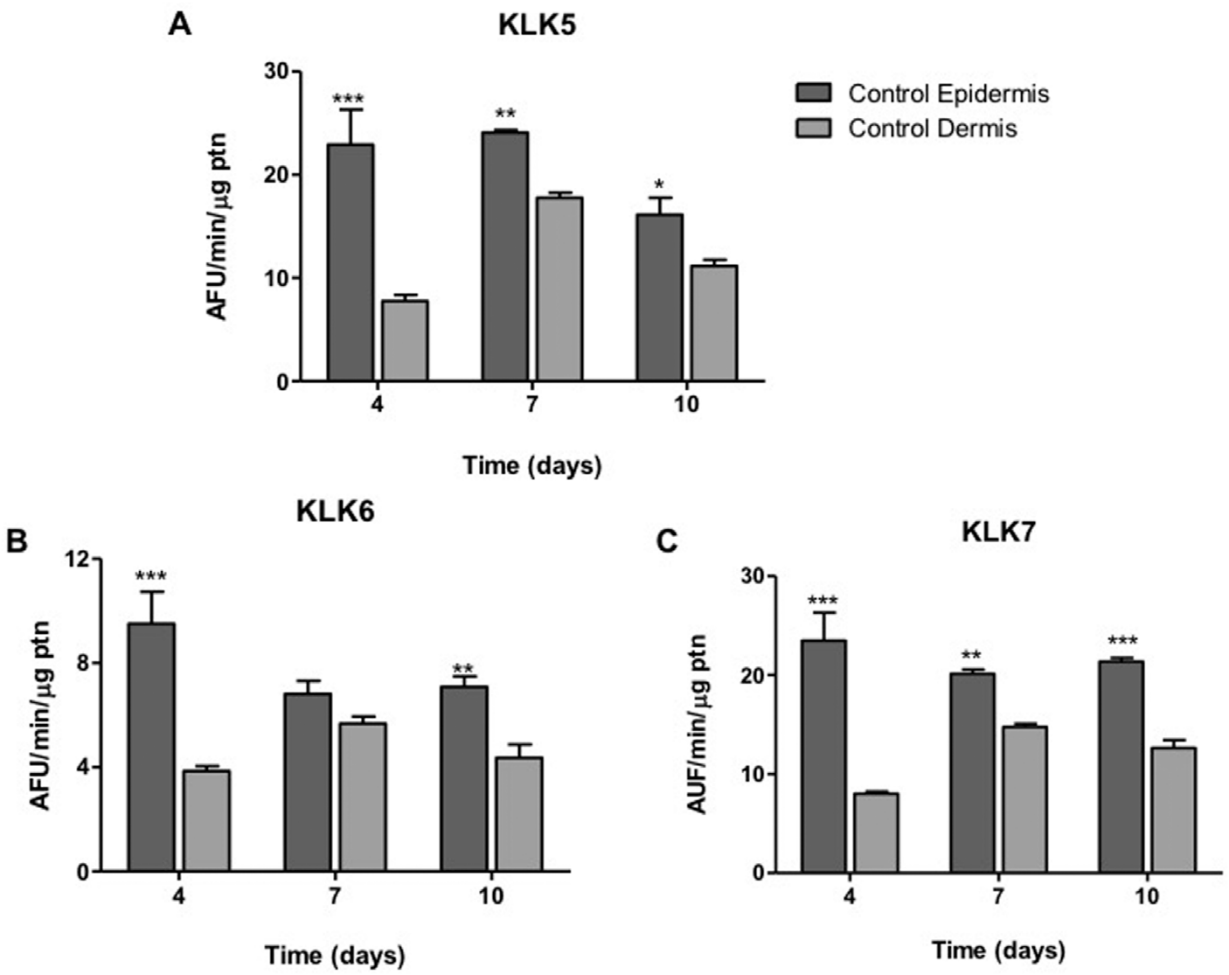
KLK activity in dermis and epidermis. The enzyme activity of **(A)** KLK5, **(B)** KLK6 and **(C)** KLK7 was detected on the hydrolysis of FRET substrates by proteins present in the extracts of epidermis (containing basal keratinocytes) and dermis (containing fibroblasts) at different periods (4, 7, 10 days) of ALI. Differences were analyzed by ANOVA + Bonferroni post-test. * (*p* < .05), ** (*p* < .01) or *** (*p* < .001), different from dermis in the same cultivation period. AFU/min/μg ptn: arbitrary fluorescence unit/minute/μg of protein in the epidermis or dermis extract.

For all KLK, at the different periods of time, except for KLK6 on day 7, it was observed a difference of at least 1.5 times between activity in the control epidermis and dermis, formed by keratinocytes and collagen with fibroblasts, respectively. The highest difference (up to a 3-fold) in KLK expression in these two layers occurred on day 4 (Figure 8). There was no difference between KLK activity in the control epidermis at different time periods.

**FIGURE 9:**
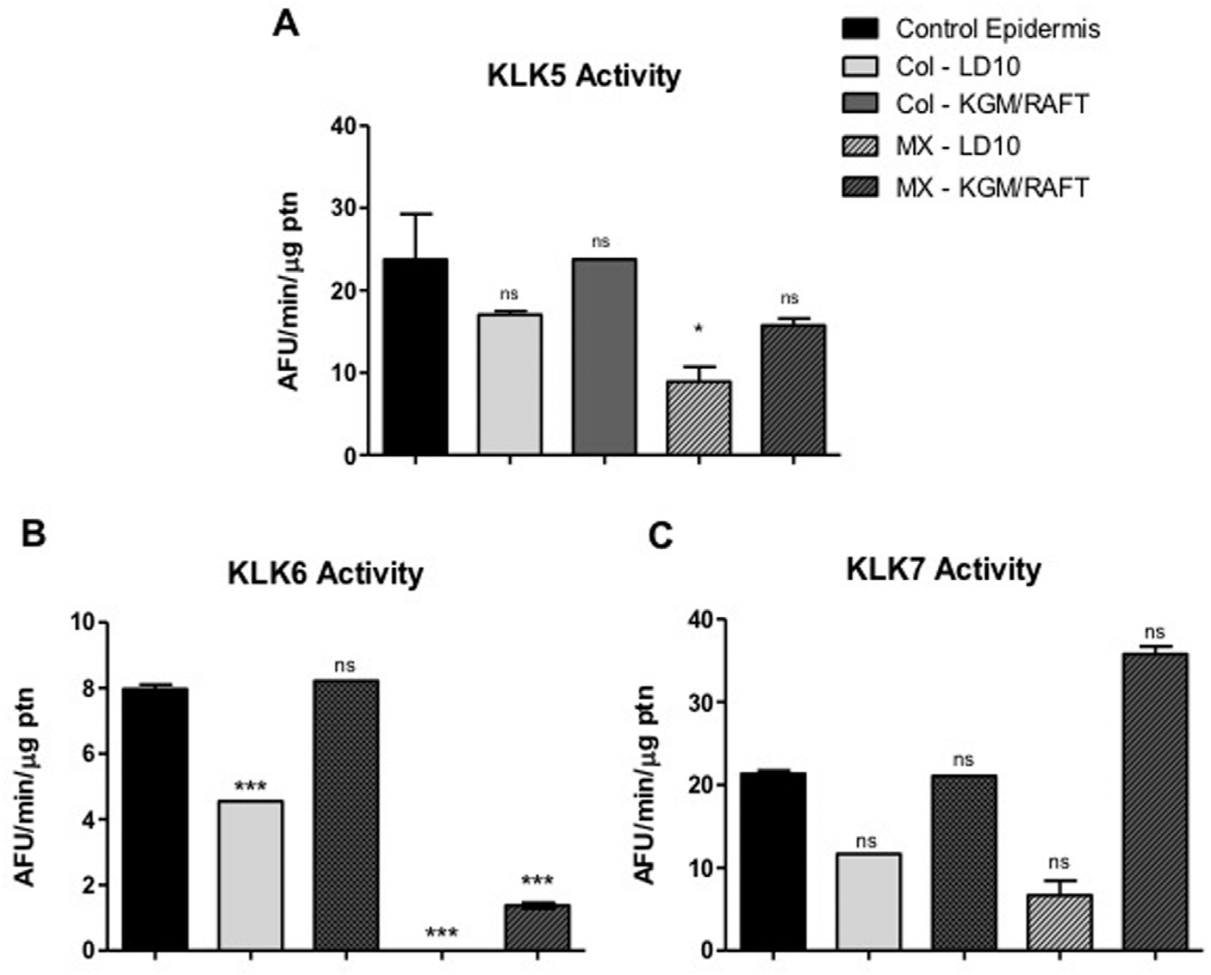
KLK activity in MSC induced to epidermal differentiation onto dermal equivalents. MSCs were cultured for 1 day in the different differentiation media or LD10 and exposed to ALI for 10 days. The enzymatic activity of **(A)** KLK5, **(B)** KLK6 and **(C)** KLK7 in cell extracts was determined on the hydrolysis of their respective substrates, in keratinocytes or MSCs cultured onto the dermal equivalent of collagen with fibroblasts or MatriXpec™. Control epidermis formed by basal keratinocytes. ns: not significant. * (*p* < .05) or *** (*p* < .001), different compared to control epidermis. KLK: tissue kallikrein. AFU/min/μg ptn: arbitrary fluorescence unit/minute/μg of protein in the epidermis extract.

Regarding the KLK5, KLK6 and KLK7 activities in the potential epidermis formed by MSC, there is no significant difference in comparison to the control epidermis composed by basal keratinocytes when grown for 1 day immersed in KGM/DM and then submitted to ALI in DM medium (Figure 9).

Specifically, KLK5 activity was 1.3-fold higher in cells induced to differentiate onto collagen and 1.8-fold higher over MatriXpec™ when compared to samples cultured in LD10 (Figure 9A). Additionally, KLK5 activity in MSC cultured onto collagen was 1.4 times higher than in cells cultured on MatriXpec™.

Even though KLK6 activity was not well detected in MSC induced to differentiate into keratinocytes onto MatriXpec™, the enzyme activity was higher in comparison to cells cultured with LD10 on the same dermal substrate (Figure 9B). Importantly, this activity was similar between MSC cultured with KGM/DM onto collagen and control epidermis, and it was 50% smaller in MSC cultured with LD10, indicating that epidermal differentiation was triggered.

KLK7 activity in differentiated MSC cultivated onto MatriXpec™ was 5.3 times higher than in cells not induced to differentiate on the same dermal support. In the collagen matrix, the activity was 1.9 times higher in cells induced to differentiate in comparison to cells cultured with LD10 during the same period. Furthermore, this activity was 2.8-fold higher in cells cultured on MatriXpec™ in relation to cells cultured on collagen considering the same period of cultivation (Figure 9C).

## Discussion

The first used dermal equivalent in research was a type I collagen plug associated with human dermal fibroblasts. *In vitro* organotypic skin models frequently use this dermal structure together primary keratinocytes induced to epidermal differentiation, which have been used for different applications, including toxicological tests (Brohem et al., 2011; Bellas et al., 2012; Catarino et al., 2018; Randall et al., 2018). Smits et al. (2017) also have shown that N/TERT keratinocyte cell lines are useful substitutes for primary human keratinocytes for both *in vitro* studies on epidermal biology and inflammatory skin disease pathogenesis.

In the present work, we have shown the epidermis reconstruction using basal keratinocytes onto the dermal equivalent constituted by collagen with fibroblasts, which displayed typical structural organization with the presence of all epidermal layers. This experiment was considered the proof of concept for the process of epidermal differentiation as it is well established in research for *in vitro* 3D skin models (Roger et al., 2019).

Despite of its feasibility for *in vitro* tests, this model is not easily applicable in the clinic, since the somatic cells can cause immunological rejection in the patient, and the use of epidermal cell lines, such as HaCaT, has been shown to be not viable for the development of an organized epidermis, in addition of having a limited capacity to synthesize the necessary lipids for the barrier formation (Boelsma et al., 1999; Schoop et al., 1999). Therefore, the potential of MSCs to transdifferentiate into keratinocytes both *in vitro* and *in vivo* have suggested their use in models of epidermis or skin barrier, which could be interesting for clinical usage (Bishai et al., 2013; Chen et al., 2015; Martin-Piedra et al., 2019; Santos et al., 2019).

Specifically, we showed that MSC from umbilical cord contributed to form a stratified organotypic culture but not a typical epithelial structure in both used dermal equivalents—collagen with fibroblasts and the decellularized matrix MatriXpec™, being the structural and chemical characteristics of dermal equivalents critical for the epidermis stratification and keratinocyte complete differentiation. In those organotypic cultures it was verified the expression of essential epidermal molecular markers such as cytokeratins (CK 5 and CK10), involucrin, filaggrin and the epidermal kallikreins (KLK), proteins that are critical to maintaining skin function and homeostasis (Kalinska et al., 2016; Evtushenko et al., 2021).

After cultivation of MSCs with DM onto the collagen plug containing dermal fibroblasts, the expression of the epidermal proteins CK14, CK10, involucrin and filaggrin was higher in comparison to cells cultured with LD10 (proliferation medium), as demonstrated by qPCR and immunofluorescence. Specifically, this increase was significant at day 7 of culture in ALI for CK14, CK10, and involucrin, and at day 10 for filaggrin compared to MSCs growth with LD10. It is interesting to verify that filaggrin, which is a marker of terminal differentiation of keratinocytes and found in the most differentiated layers of epidermis, was detected after the peak of CK10 expression, maintaining a biological identity with the *in vivo* phenomenon. In this sense, our group has already demonstrated the increased expression of these markers when MSCs were two-dimensionally grown with KSFM, a medium for keratinocyte cultivation supplemented with 5 ng/mL EGF, being the 7th day of culture decisive for the expression of epidermal markers such as CK10 and involucrin (Santos et al., 2019).

These results corroborate Martin-Piedra et al. (2019), which analyzed the expression of different epidermal markers when MSCs derived from the adipose tissue, dental pulp, umbilical cord and bone marrow were differentiated into keratinocytes in organotypic cultures *in vitro* and *in vivo*, after 21 days of ALI. The authors showed that the cultivation of MSC from umbilical cord and bone marrow resulted in an increased number of epidermal layers compared to other cellular sources, in addition to a moderate expression of CK10 and filaggrin *in vitro*. These results evidenced that protocols for 3D epidermal transdifferentiation of MSCs, despite not inducing an essential change from the mesenchymal to epithelial phenotype, were able to trigger epidermal differentiation *in vitro*, which effectively occurred after *in vivo* cell transplantation. Also, the expression of those proteins was increased by prolonged exposure times in ALI (Martin-Piedra et al., 2019). Another important finding of these authors was to show that MSCs from umbilical cord did not express human leukocyte antigen (HLA) class 1 and 2 in culture both *in vitro* and *in vivo*, unlike MSCs from bone marrow, which reduce rejection of cells or tissue grafts in allogeneic recipients (Kulus et al., 2021). Regarding perspectives for clinical use, MSCs from umbilical cord are preferred, since they remain poorly immunogenic after epidermal induction, evidencing the significance of these cells for future therapeutic trials involving the human skin.

In order to go forward in the search for a dermal equivalent able to contribute and sustain MSC epidermal differentiation, we have used the commercial support MatriXpecTM. Histological analysis suggested the possibility of the formation of connective tissue, which is corroborated by the fact that the fibroblasts, the main cells in this tissue, are also of mesenchymal origin (Chang et al., 2014). Interestingly, despite the no typical epidermal phenotype of MSC in this condition, cells were able to express CK10 and involucrin, as assessed by immunofluorescence analysis, which did not occur in organotypic cultures maintained with LD10.

We have also shown that MSC cultured with LD10 (proliferation medium) onto the MatriXpec presented an invasive phenotype toward the dermal equivalent, suggesting that the basal culture condition may preserve the stem capacity of MSC and it was critical to the fate of cells. The unique and distinct behavior of the MSC cultivated in LD10 in the two used matrices, which resulted from distinct production processes, might be related to preservation of the extracellular matrix (ECM) 3D architecture (Kumar et al., 2021), being well known that the extraction process of ECM, involving chemical, enzymatic, physical and/or mechanical methods, may disrupt key ECM proteins such as the collagen (Kamalvand et al., 2021), favoring cell migration and invasion into the dermal equivalent. Also, it is important to consider that the concentration of the gel in the dermal supports is not necessarily the same and may offer different resistance for cell migration and conditions for proliferation/differentiation.

Particularly, studies involving MSCs induced to epidermal differentiation on dermal matrices also showed a migratory profile of the cells toward the dermal equivalent (Schneider et al., 2008; Schneider et al., 2010). These authors suggested that the expression of vimentin in MSCs from umbilical cord might contribute to the observed tissue organization and the healing process, by remodeling the ECM, but not exactly by differentiating into keratinocytes (Schneider et al., 2008; Schneider et al., 2010; Martin-Piedra et al., 2019).

The presence of EGF in the culture medium has been also related to this invasive behavior of MSCs into the dermal equivalents. It is known that this growth factor promotes the differentiation and migration of keratinocytes, in addition to the formation of granulation tissue, which are important events for wound healing process (Oda et al., 2005; Li et al., 2021). Regarding the EGF involvement in MSC migration into the dermal equivalent, the present results corroborate Schneider et al. (2010), which demonstrated that the culturing of MSC-based organotypic cultures with 20 ng/mL EGF for 21 days induced epidermal differentiation, caused progressive contraction of the collagen plug and resulted in the formation of a cluster of MSC inside the dermal equivalent. Similar results were found by Kao (2021) when culturing MSCs from human umbilical cord with LD10 on a scaffold composed of platelet-rich plasma gel and exposed to ALI. In addition to the proliferation and migration of cells towards the scaffold, the authors showed that MSCs expressed pan-cytokeratin and the nuclear marker p63 found in epidermal precursors, showing that the composition of the support is decisive to the phenotype that these cells will acquire. Even though the MSC phenotype in 3D skin models has been correlated to EGF presence and concentration, further studies are necessary to clear the exact role and to optimize its concentration in order to produce a mimetic MSC-based epidermis and full skin.

Previous studies have shown that MSCs can migrate into a specific substrate or tissue in response to chemotactic factors including inflammatory cytokines, growth factors, and chemokines produced by the injured tissue (Spaeth et al., 2012). In this respect, the stem cell paracrine hypothesis postulates the paracrine action of transplanted stem cells by secreting soluble and insoluble factors into the extracellular space (Tögel et al., 2005; Cai et al., 2016; Danieli et al., 2016; Gnecchi et al., 2016). Nevertheless, the full extent of MSCs and their paracrine effects, particularly *via* extracellular vesicles (EV), are not completely understood but it has been shown promising results in the control of inflammation, acceleration of skin cell migration and proliferation, controlling wound scarring, improvement of angiogenesis, and even ameliorating signs of skin aging (Ferreira and Gomes, 2018).

Because of the unexpected phenotype and invasiveness of MSC grown onto the dermal equivalents, we have investigated the commitment of these cells to epidermal differentiation based on the expression of molecules that are involved in epithelial-mesenchymal transition (EMT) and in its reverse process mesenchymal–epithelial transition (MET), which is employed to generate epithelia at different developmental stages.

During MET, mesenchymal cells progressively establish apical basal polarity through an evolutionarily conserved group of proteins and well-defined mechanisms (Rodriguez-Boulan and Macara, 2014). In health situations, epithelial cells are in direct communication with the stromal compartment *via* an organized structure and are arranged with adjacent cells *via* tight and gap junctions. These connections not only act for communication and limit both cell proliferation and migration, but also establish a cell polarity. Specifically, the tight junctions are constructed upon homotypic binding of epithelial-cadherins (Wells et al., 2008). In this sense, a critical molecular feature of EMT is the downregulation of e-cadherin, whereas in MET, e-cadherin is up-regulated. On the other hand, vimentin is one of EMT protein markers, which is present in mesenchymal cells and involved in cancer progression (Kalluri and Weinberg, 2009; Zeisberg and Neilson, 2009).

Specifically, quantitative PCR results showed that e-cadherin was up-regulated in MSC induced to differentiate onto both dermal equivalents (collagen or MatriXpec) and the expression was increased over the time of ALI. Interestingly, e-cadherin expression was higher in MSC cultivated onto MatrixPec in comparison to collagen, however, in skin assembled with keratinocytes it was observed the opposite. Obviously, the expression of E-cadherin was much higher in keratinocytes-reconstructed skins in comparison to MSC, however, combining E-cadherin up regulation and vimentin down regulation in MSC induced to epidermal differentiation, new insights to understand the behavior of these cells, being possible to suggest that MSC were committed to epidermal differentiation in the current model.

In addition to the histological analyses and the expression of epidermal markers by qPCR and immunofluorescence, an important biochemical evaluation performed in the present work was the quantification of the activity of KLK, which are enzymes involved in the physiology of skin, and related to the terminal differentiation of keratinocytes. We have already demonstrated the activity of these enzymes in the process of MSC transdifferentiation into keratinocytes in a 2D model, showing that they can be used to ease and rapidly monitor the commitment to epidermal differentiation process (Santos et al., 2019). Regarding the 3D model, significant differences in KLK activity were observed between the control epidermis and dermis, although the activity did not vary significantly with the time of exposure to ALI. This result demonstrates the selectivity of KLK activity in keratinocytes, not being significantly detected in the fibroblasts present in the dermal equivalent.

The activity of KLK in the potential epidermis formed by MSCs in both dermal equivalents was similar to the one found in the control epidermis. Furthermore, KLK activity was about 2 to 3 times higher when MSCs are induced to epidermal differentiation, compared to cells cultured with LD10, in all used dermal equivalents, which corroborates the data obtained during the two-dimensional differentiation of these cells (Santos et al., 2019). These data clearly indicate the commitment of MSCs with the process of epidermal differentiation onto the used dermal equivalents, and suggest that these cells have the enzymatic machinery necessary for the differentiation.

Collectively, the results suggest that the use of the type I collagen seems to be more adequate to understand how chemical factors can influence the differentiation of these cells, since the composition of the decellularized matrix, which is derived from a biological tissue, may vary between the experiments and affect the reproducibility.

It is important to highlight that the previously reported attempt to produce a MSC-based epidermis failed to show the specific expression of suprabasal markers such as CK10, involucrin, and filaggrin, for example. Most studies have shown only pan-cytokeratin and/or p63 presence (Schneider et al., 2008) and barely CK10 or filaggrin (Martin-Piedra et al., 2019) in MSC induced to differentiate into keratinocytes on dermal equivalents. Additionally, despite the absence of a typical stratified epithelial phenotype, MSC incorporation in 3D skin substitutes may result in barrier function reestablishment, since important proteins are expressed such as filaggrin, besides to their role in wound healing process, which may accelerate the tissue repair.

## Data Availability

The original contributions presented in the study are included in the article/Supplementary Material, further inquiries can be directed to the corresponding author.

## References

[B1] Angelo P. F. Lima A. R. Alves F. M. Blaber S. I. Scarisbrick I. A. Blaber M. (2006). Substrate specificity of human kallikrein 6. J. Biol. Chem. 281, 3116–3126. 10.1074/jbc.M510096200 16321973

[B2] Bellas E. Seiberg M. Garlick J. Kaplan D. L. (2012). *In vitro* 3D full-thickness skin-equivalent tissue model using silk and collagen biomaterials. Macromol. Biosci. 12, 1627–1636. 10.1002/mabi.201200262 23161763 PMC3724336

[B3] Bian D. Wu Y. Song G. Azizi R. Zamani A. (2022). The application of mesenchymal stromal cells (MSCs) and their derivative exosome in skin wound healing: A comprehensive review. Stem Cell. Res. Ther. 13, 24. 10.1186/s13287-021-02697-9 35073970 PMC8785459

[B4] Bishai I. E. M. El Ansary M. S. Shaheen N. M. H. Farid R. J. (2013). Mesenchymal stem cell separation from Wharton’s jelly and its differentiation into keratinocytes. Comp. Clin. Path. 22, 547–553. 10.1007/s00580-013-1702-z

[B5] Boelsma E. Verhoeven M. C. H. Ponec M. (1999). Reconstruction of a human skin equivalent using a spontaneously transformed keratinocyte cell line (HaCaT). J. Invest. Dermatol. 112, 489–498. 10.1046/j.1523-1747.1999.00545.x 10201534

[B6] Brohem C. A. da Silva Cardeal L. B. Tiago M. Soengas M. S. de Moraes Barros S. B. Maria-Engler S. S. (2011). Artificial skin in perspective: Concepts and applications. Pigment. Cell. Melanoma Res. 24, 35–50. 10.1111/j.1755-148X.2010.00786.x 21029393 PMC3021617

[B7] Cai M. Shen R. Song L. Lu M. Wang J. Zhao S. (2016). Bone marrow mesenchymal stem cells (BM-MSCs) improve heart function in swine myocardial infarction model through paracrine effects. Sci. Rep. 6, 28250. 10.1038/srep28250 27321050 PMC4913323

[B8] Capilla-González V. Herranz-Pérez V. Sarabia-Estrada R. Kadri N. Moll G. (2022). Editorial: Mesenchymal stromal cell therapy for regenerative medicine. Front. Cell. Neurosci. 16, 932281. 10.3389/fncel.2022.932281 35693887 PMC9179645

[B9] Catarino C. M. do Nascimento Pedrosa T. Pennacchi P. C. de Assis S. R. Gimenes F. Consolaro M. E. L. (2018). Skin corrosion test: A comparison between reconstructed human epidermis and full thickness skin models. Eur. J. Pharm. Biopharm. 125, 51–57. 10.1016/j.ejpb.2018.01.002 29317274

[B10] Chang Y. Li H. Guo Z. (2014). Mesenchymal stem cell-like properties in fibroblasts. Cell. Physiol. biochem. 34, 703–714. 10.1159/000363035 25171291

[B11] Chen D. Hao H. Tong C. Liu J. Dong L. Ti D. (2015). Transdifferentiation of umbilical cord–derived mesenchymal stem cells into epidermal-like cells by the mimicking skin microenvironment. Int. J. Low. Extrem. Wounds 14, 136–145. 10.1177/1534734615569913 25700709

[B12] Danieli P. Malpasso G. Ciuffreda M. C. Gnecchi M. (2016). Testing the paracrine properties of human mesenchymal stem cells using conditioned medium. Methods Mol. Biol. 1416, 445–456. 10.1007/978-1-4939-3584-0_26 27236688

[B13] Evtushenko N. A. Beilin A. K. Kosykh A. V. Vorotelyak E. A. Gurskaya N. G. (2021). Keratins as an inflammation trigger point in epidermolysis bullosa simplex. Int. J. Mol. Sci. 22, 12446. 10.3390/ijms222212446 34830328 PMC8624175

[B14] Fernandes M. T. P. dos Santos J. F. Freitas B. L. Reigado G. R. Antunes F. Tessarollo N. G. (2022). Reporter system controlled by the involucrin promoter as a tool to follow epidermal differentiation. Biochimie 201, 33–42. 10.1016/j.biochi.2022.06.014 35792308

[B15] Ferreira A. da F. Gomes D. A. (2018). Stem cell extracellular vesicles in skin repair. Bioengineering 6, 4. 10.3390/bioengineering6010004 30598033 PMC6466099

[B16] Gnecchi M. Danieli P. Malpasso G. Ciuffreda M. C. (2016). Paracrine mechanisms of mesenchymal stem cells in tissue repair, Methods Mol. Biol. 1416, 123–146. 10.1007/978-1-4939-3584-0_7 27236669

[B18] Iljas J. D. Röhl J. McGovern J. A. Moromizato K. H. Parker T. J. Cuttle L. (2021). A human skin equivalent burn model to study the effect of a nanocrystalline silver dressing on wound healing. Burns 47, 417–429. 10.1016/j.burns.2020.07.007 32830005

[B19] Kalinska M. Meyer-Hoffert U. Kantyka T. Potempa J. (2016). Kallikreins – the melting pot of activity and function. Biochimie 122, 270–282. 10.1016/j.biochi.2015.09.023 26408415 PMC4747678

[B54] Kalluri R. Weinberg R. A. (2009). The basics of epithelial-mesenchymal transition. J. Clin. Invest. 119, 1420–1428. 10.1172/JCI39104 19487818 PMC2689101

[B20] Kamalvand M. Biazar E. Daliri-Joupari M. Montazer F. Rezaei-Tavirani M. Heidari-Keshel S. (2021). Design of a decellularized fish skin as a biological scaffold for skin tissue regeneration. Tissue Cell. 71, 101509. 10.1016/j.tice.2021.101509 33621947

[B21] Kamolz L. P. Kolbus A. Wick N. Mazal P. R. Eisenbock B. Burjak S. (2006). Cultured human epithelium: Human umbilical cord blood stem cells differentiate into keratinocytes under *in vitro* conditions. Burns 32, 16–19. 10.1016/j.burns.2005.08.020 16368194

[B22] Kao C. H. (2021). Application of concentrated growth factors membrane for human umbilical cord wharton’s jelly mesenchymal stem cell differentiation towards keratinocytes. Separations 8, 61. 10.3390/separations8050061

[B23] Kulus M. Sibiak R. Stefańska K. Zdun M. Wieczorkiewicz M. Piotrowska-Kempisty H. (2021). Mesenchymal stem/stromal cells derived from human and animal perinatal tissues—origins, characteristics, signaling pathways, and clinical trials. Cells 10, 3278. 10.3390/cells10123278 34943786 PMC8699543

[B24] Kumar N. Kumar V. Purohit S. Gangwar A. K. Shrivastava S. Maiti S. K. (2021). Decellularization of skin tissue, Adv. Exp. Med. Biol. 1345, 165–191. 10.1007/978-3-030-82735-9_15 34582023

[B25] Li B. Tang H. Bian X. Ma K. Chang J. Fu X. (2021). Calcium silicate accelerates cutaneous wound healing with enhanced re-epithelialization through EGF/EGFR/ERK-mediated promotion of epidermal stem cell functions. Burn. Trauma 9, tkab029. 10.1093/burnst/tkab029 PMC8484206 34604395

[B26] Loura L. Prieto M. (2011). FRET in membrane biophysics: An overview. Front. Physiol. 2, 82. 10.3389/fphys.2011.00082 22110442 PMC3216123

[B27] Martin-Piedra M. Alfonso-Rodriguez C. A. Zapater A. Durand-Herrera D. Chato-Astrain J. Campos F. (2019). Effective use of mesenchymal stem cells in human skin substitutes generated by tissue engineering. Eur. Cells Mat. 37, 233–249. 10.22203/eCM.v037a14 30924522

[B28] Nourian Dehkordi A. Mirahmadi Babaheydari F. Chehelgerdi M. Raeisi Dehkordi S. (2019). Skin tissue engineering: Wound healing based on stem-cell-based therapeutic strategies. Stem Cell. Res. Ther. 10, 111. 10.1186/s13287-019-1212-2 30922387 PMC6440165

[B29] Nunes V. A. Cavaçana N. Canovas M. Strauss B. E. Zatz M. (2007). Stem cells from umbilical cord blood differentiate into myotubes and express dystrophin *in vitro* only after exposure to *in vivo* muscle environment. Biol. Cell. 99, 185–196. 10.1042/BC20060075 17166095

[B30] Oda K. Matsuoka Y. Funahashi A. Kitano H. (2005). A comprehensive pathway map of epidermal growth factor receptor signaling. Mol. Syst. Biol. 1, 2005.0010. 10.1038/msb4100014 PMC1681468 16729045

[B32] Randall M. J. Jüngel A. Rimann M. Wuertz-Kozak K. (2018). Advances in the biofabrication of 3D skin *in vitro*: Healthy and pathological models. Front. Bioeng. Biotechnol. 6, 154. 10.3389/fbioe.2018.00154 30430109 PMC6220074

[B55] Rodriguez-Boulan E. Macara I. G. (2014). Organization and execution of the epithelial polarity programme. Nat. Rev. Mol. Cell Biol. 15, 225–242. 10.1038/nrm3775 24651541 PMC4211427

[B33] Roger M. Fullard N. Costello L. Bradbury S. Markiewicz E. O’Reilly S. (2019). Bioengineering the microanatomy of human skin. J. Anat. 234, 438–455. 10.1111/joa.12942 30740672 PMC6422806

[B35] Santos J. F. Borçari N. R. da Silva Araújo M. Nunes V. A. (2019). Mesenchymal stem cells differentiate into keratinocytes and express epidermal kallikreins: Towards an *in vitro* model of human epidermis. J. Cell. Biochem. 120, 13141–13155. 10.1002/jcb.28589 30891818

[B36] Schneider C. A. Rasband W. S. Eliceiri K. W. (2012). NIH image to ImageJ: 25 years of image analysis. Nat. Methods 9, 671–675. 10.1038/nmeth.2089 22930834 PMC5554542

[B37] Schneider R. K. M. Neuss S. Stainforth R. Laddach N. Bovi M. Knuechel R. (2008). Three-dimensional epidermis-like growth of human mesenchymal stem cells on dermal equivalents: Contribution to tissue organization by adaptation of myofibroblastic phenotype and function. Differentiation 76, 156–167. 10.1111/j.1432-0436.2007.00204.x 17634073

[B38] Schneider R. K. Püllen A. Kramann R. Bornemann J. Knüchel R. Neuss S. (2010). Long-term survival and characterisation of human umbilical cord-derived mesenchymal stem cells on dermal equivalents. Differentiation 79, 182–193. 10.1016/j.diff.2010.01.005 20153102

[B39] Schoop V. M. Fusenig N. E. Mirancea N. (1999). Epidermal organization and differentiation of HaCaT keratinocytes in organotypic coculture with human dermal fibroblasts. J. Invest. Dermatol. 112, 343–353. 10.1046/j.1523-1747.1999.00524.x 10084313

[B40] Smith C. J. Parkinson E. K. Yang J. Pratten J. O’Toole E. A. Caley M. P. (2022). Investigating wound healing characteristics of gingival and skin keratinocytes in organotypic cultures. J. Dent. 125, 104251. 10.1016/j.jdent.2022.104251 35961474

[B41] Smits J. P. H. Niehues H. Rikken G. van Vlijmen-Willems I. M. J. J. van de Zande G. W. H. J. F. Zeeuwen P. L. J. M. (2017). Immortalized N/TERT keratinocytes as an alternative cell source in 3D human epidermal models. Sci. Rep. 7, 11838. 10.1038/s41598-017-12041-y 28928444 PMC5605545

[B42] Spaeth E. L. Kidd S. Marini F. C. (2012). “Tracking inflammation-induced mobilization of mesenchymal stem cells,” in Stem cell mobilization (Totowa, NJ: Humana Press), 173–190. 10.1007/978-1-61779-943-3_15 22890932

[B43] Stefańska K. Ożegowska K. Hutchings G. Popis M. Moncrieff L. Dompe C. (2020). Human wharton’s jelly—cellular specificity, stemness potency, animal models, and current application in human clinical trials. J. Clin. Med. 9, 1102. 10.3390/jcm9041102 32290584 PMC7230974

[B44] Steffens D. Mathor M. B. Santi B. T. Luco D. P. Pranke P. (2015). Development of a biomaterial associated with mesenchymal stem cells and keratinocytes for use as a skin substitute. Regen. Med. 10, 975–987. 10.2217/rme.15.58 26542841

[B45] Stewart C. E. Torr E. E. Mohd Jamili N. H. Bosquillon C. Sayers I. (2012). Evaluation of differentiated human bronchial epithelial cell culture systems for asthma research. J. Allergy 2012, 943982. 10.1155/2012/943982 PMC3263641 22287976

[B46] Sudo T. Iwaya T. Nishida N. Sawada G. Takahashi Y. Ishibashi M. (2013). Expression of mesenchymal markers vimentin and fibronectin: The clinical significance in esophageal squamous cell carcinoma. Ann. Surg. Oncol. 20, 324–335. 10.1245/s10434-012-2418-z 22644514

[B47] Tam K. Cheyyatraviendran S. Venugopal J. Biswas A. Choolani M. Ramakrishna S. (2014). A nanoscaffold impregnated with human wharton’s jelly stem cells or its secretions improves healing of wounds. J. Cell. Biochem. 115, 794–803. 10.1002/jcb.24723 24265214

[B48] Toai T. C. Thao H. D. Gargiulo C. Thao N. P. Thuy T. T. T. Tuan H. M. (2011). *In vitro* culture of keratinocytes from human umbilical cord blood mesenchymal stem cells: The saigonese culture. Cell. Tissue Bank. 12, 125–133. 10.1007/s10561-010-9174-8 20349146

[B49] Tögel F. Hu Z. Weiss K. Isaac J. Lange C. Westenfelder C. (2005). Administered mesenchymal stem cells protect against ischemic acute renal failure through differentiation-independent mechanisms. Am. J. Physiol. Physiol. 289, F31–F42. 10.1152/ajprenal.00007.2005 15713913

[B50] Wang H. Hung S. Peng S. Huang C. Wei H. Guo Y. (2004). Mesenchymal stem cells in the wharton’s jelly of the human umbilical cord. Stem Cells 22, 1330–1337. 10.1634/stemcells.2004-0013 15579650

[B51] Wang X. W. Wang J. J. Gutowska-Owsiak D. Salimi M. Selvakumar T. A. Gwela A. (2017). Deficiency of filaggrin regulates endogenous cysteine protease activity, leading to impaired skin barrier function. Clin. Exp. Dermatol. 42, 622–631. 10.1111/ced.13113 28556377

[B56] Wells A. Yates C. Shepard C. R. (2008). E-cadherin as an indicator of mesenchymal to epithelial reverting transitions during the metastatic seeding of disseminated carcinomas. Clin. Exp. Metastasis 25, 621–628. 10.1007/s10585-008-9167-1 18600305 PMC2929356

[B52] Xie H. Sun L. Zhang L. Liu T. Chen L. Zhao A. (2016). Mesenchymal stem cell-derived microvesicles support *ex vivo* expansion of cord blood-derived CD34 + cells. Stem Cells Int. 2016, 6493241. 10.1155/2016/6493241 27042183 PMC4799819

[B53] Yi S. Ding F. Gong L. Gu X. (2017). Extracellular matrix scaffolds for tissue engineering and regenerative medicine. Curr. Stem Cell. Res. Ther. 12, 233–246. 10.2174/1574888X11666160905092513 27593448

[B57] Zeisberg M. Neilson E. G. (2009). Biomarkers for epithelial-mesenchymal transitions. J. Clin. Invest. 119, 1429–1437. 10.1172/JCI36183 19487819 PMC2689132

